# Do consumer voices in health‐care citizens’ juries matter?

**DOI:** 10.1111/hex.12397

**Published:** 2015-09-28

**Authors:** Rachael Krinks, Elizabeth Kendall, Jennifer A. Whitty, Paul A. Scuffham

**Affiliations:** ^1^ School of Human Services and Social WorkMenzies Health Institute QldGriffith UniversityLoganQldAustralia; ^2^ Centre of National Research on Disability and RehabilitationMenzies Health Institute QldGriffith UniversityLoganQldAustralia; ^3^ School of PharmacyFaculty of Health and Behavioural SciencesThe University of QueenslandBrisbaneQldAustralia; ^4^ Centre for Applied Health EconomicsSchool of MedicineGriffith UniversityBrisbaneQldAustralia; ^5^ Director of the Centre for Applied Health Economics and Director of the Population and Social Health Research ProgramMenzies Health Institute QldGriffith UniversityBrisbaneQldAustralia

**Keywords:** citizen deliberation, citizens’ juries, consumer voice, decision‐making, engagement, health‐care priorities, participation, patient and public involvement

## Abstract

**Background:**

There is widespread agreement that the public should be engaged in health‐care decision making. One method of engagement that is gaining prominence is the citizens’ jury, which places *citizens* at the centre of the deliberative process. However, little is known about how the jury process works in a health‐care context. There is even less clarity about how *consumer* perspectives are heard within citizens’ juries and with what consequences.

**Objectives:**

This paper focuses on what is known about the role of consumer voices within health‐care citizens’ juries, how these voices are heard by jurors and whether and in what ways the inclusion or exclusion of such voices may matter.

**Results:**

Consumer voices are not always included in health‐care citizens’ juries. There is a dearth of research on the conditions under which consumer voices emerge (or not), from which sources and why. As a result, little is known about what stories are voiced or silenced, and how such stories are heard by jurors, with what consequences for jurors, deliberation, decision‐makers, policy and practice.

**Discussion and Conclusion:**

The potential role of consumer voices in influencing deliberations and recommendations of citizens’ juries requires greater attention. Much needed knowledge about the nuances of deliberative processes will contribute to an assessment of the usefulness of citizens’ juries as a public engagement mechanism.

## Do consumer voices in health‐care citizens’ juries matter?

Citizens’ juries are increasingly being used by governments as a deliberative method of engaging citizens in health‐care priority setting.^1^, ^2^, ^3^, ^4^ Citizens’ juries are seen as a useful mechanism for engaging the public because they elicit the informed views of a group of representative citizens in a systematic way that connects ‘health‐care policy with the concerns of the broader community’.^5^ Early evidence indicates that ordinary citizens are up to the task of absorbing, analysing, deliberating and deciding on complex technical, health, scientific and ethical issues.^3^, ^6^, ^7^, ^8^, ^9^ However, knowledge about the use of deliberative processes in the health context remains limited because the ‘use of deliberative approaches has not been widely documented, particularly by health agencies in Australia’.^10^, ^11^, ^12^, ^13^

One aspect of citizens’ juries that has yet to be investigated is the lens that is brought to the deliberation by the participants (jurors) themselves. In‐depth research on the perspectives of jurors is scarce, leaving us bereft of knowledge about how juries are experienced. The premise of citizens’ juries is the engagement of informed but unbiased, representative views in health policy decision making. However, all jurors are health consumers,^1^ and inevitably (perhaps desirably) bring their own experience to the process. The role of consumer voices in health‐care citizens’ juries, how these voices are heard by jurors, and with what consequences for the deliberative process, remains unclear.

In this paper, we discuss the potential for consumer voices to influence the supposed neutrality of a citizens’ jury, which may be problematic given that juries are constructed with the intention of eliciting views that reflect those of the general population. We first introduce the citizens’ jury as a deliberative engagement method and then clarify what is known about the role of consumer voices and their influence on deliberation within health‐care citizen juries. We argue that the distinction between a citizen and a consumer may not be sustainable in practice, but may also unfairly marginalize individuals and groups who make sense of the world by sharing, listening to and comparing real‐life health experiences. Crucially, the absence of consumer voices may contest the legitimacy of a deliberative public engagement process.

## What are citizens’ juries?

The citizens’ jury model stems in part from Germany's deliberative ‘planning cells’ which have been part of the deliberative policymaking landscape in Germany since the 1960s.^6^, ^15^ Citizens’ juries were introduced in the United States in 1974 to address what was seen as a ‘democratic deficit’.^11^, ^15^, ^16^, ^17^ Citizens’ juries (and citizens’ panels or councils in which the same jurors engage in meetings over longer periods of time) have been held in a diverse range of countries including Canada and India.^6^, ^7^, ^18^, ^19^ However, the majority of citizens’ juries, including those on health topics, have been convened in the USA and the UK.

In most cases, a citizens’ jury is a group of about 12–24 citizens, selected using stratified random sampling, who deliberate on a public policy issue.^2^, ^6^, ^7^, ^8^ The jurors are invited to meet for (usually) 3–5 days to hear, question, challenge and clarify expert witness testimony from a range of perspectives. They are assisted by a facilitator to deliberate until they form a consensus or vote on their preferred solution.^3^, ^6^ Jurors’ recommendations are summarized in a report that is subsequently presented to relevant authorities.^2^, ^6^, ^16^ In a health‐care context, expert witnesses may include clinicians, policymakers and health economists as well as ‘consumer’ witnesses who provide testimony about their personal experiences of the health issue/service or treatment under deliberation.^1^ However, expert witnesses or jurors may also express a ‘consumer’ voice if they refer to their own experiences, or that of family and friends, in relation to a health issue.

## The role of consumer perspectives in public deliberation

Citizens’ juries place *citizens*, not consumers or lobby groups, at the centre of the deliberative process.^8^, ^20^ According to Degeling *et al*., jurors are expected to be ‘disinterested members of the public (*citizens)* …[rather] than experienced ‘service‐users’ (patients or *consumers*)’ (original emphasis, Ref. 4). Jurors are generally screened and selected by organizers to identify ‘ordinary citizens with no particular axe to grind’ 17 who can ‘examine the issue without a vested interest’.^21^ Consumer voices are generally expected to only arise from consumer *witnesses* if they have been included. Whether the ‘analytical distinction’ between citizens and consumers is shared by the jurors themselves and whether such a distinction can be sustained in practice in a real‐life citizen jury are unclear.^21^ Implicit and explicit findings from recent scoping and systematic reviews of the citizens’ jury literature have called for more clarity about the role of jurors and the ‘respective legitimacy’ of those recruited to deliberate.^4^, ^22^, ^23^

Strong critiques of categories of ‘consumer’, ‘citizen’ and ‘expert’, issues of representativeness and marginalization of subjective voices in public deliberation exist but other than occasional exceptions^24^, ^25^, ^26^ in the main they are theoretical undertakings not (or only loosely) based on empirical work.^27^, ^28^, ^29^, ^30^, ^31^, ^32^, ^33^, ^34^, ^35^ The specific question of how consumer voices in citizens’ juries *are heard by jurors* and with *what consequences* has not received much empirical attention.

### Consumer voice as expert testimony – consumer witnesses

Despite the need for jurors to be exposed to all relevant perspectives, consumer witnesses are rarely invited to give testimony. The Jefferson Center in the USA has held a number of citizens’ juries on health‐care topics. According to reports published on their website, consumer witnesses are generally not included in these juries.^36^, ^37^ More recently, a Canadian demonstration project seeking the public's priorities for Ontario's health system asked jurors to develop ‘nine personae, representing a variety of health users’ to help ‘panellists consider how their recommendations would affect different demographic groups’. This method of simulating consumer voices, instead of simply asking consumers to appear as witnesses, may have misrepresented reality.^19^ Two recent citizens’ juries deliberating health‐care topics in Australia also failed to include consumer witnesses despite the relevance and importance of the issues to consumers (G Mooney, personal communication 29 May 2012 and unpublished report 2012).^38^

When consumers have been included as expert witnesses, it is often unclear how they were recruited and selected and how the issue of their representativeness was handled. Given that jurors usually have a maximum of 3 days together and receive a large volume of information from many witnesses during that time, it would be impossible to include the type of diversity required to adequately represent experiences. Even consumers ‘with identical needs may ‘experience’ the same service’ differently, leading to different evidence being presented to the jury.^34^

Consumers in the UK have raised questions about how consumer witness evidence is ‘handled, weighed and valued’ in comparison with the potentially more influential voices of ‘health professionals, health economists’ and others.^39^ The suggestion that there may be inequality between how consumer and specialist witness testimony is heard by jurors was also noted in a Welsh citizens’ jury when a consumer witness complained that ‘her evidence had been down‐graded as she was appearing in her role as a service user’ and that she had not received equal status to the professionals in terms of time and opportunity for questions.^40^

However, a more recent study concluded that ‘prominent recommendations were made on the basis’ of consumer witness evidence,^41^ attesting to the importance of this knowledge. However, the researchers also reported that ‘each juror drew on their personal experience’ in a way that was ‘crucial to the jury's deliberations’.^41^ The authors provided evidence (quotations) of jurors sharing consumer experiences of their own and that of family members,^41^ showing the potential for different consumer voices to interact in the jury context. The study did not, however, examine the way in which various voices were heard and assessed by jurors, which, if any, were ignored, marginalized or discarded, and which were influential on jurors’ deliberations.

### Consumer voice in the jury – consumer stories shared by jurors themselves

In‐depth empirical investigation about *the jurors’ consumer voices*, and the *consequences* of these voices for the jury, the verdict and any subsequent policy or practice, is scarce. There is evidence of implicit and explicit expectations that citizen jurors should leave their health consumer ‘baggage’ at the door, but this theoretical distinction between citizen and consumer identities may not be sustainable in practice. Facilitators of citizens’ juries overseas and in Australia have made it clear to jurors ‘that personal narratives’ are ‘not always welcome’.^20^ In Australia, jurors have explicitly been directed to not ‘talk directly of their cancer’ or personal health experiences and to not bring ‘their own personal baggage with them’.^3^ The view of the late Gavin Mooney, an Australian health economist and citizens’ jury expert who has facilitated the most health‐care juries in Australia to date, was that ‘the role of the jurors is citizens not consumers and I draw quite a strong line between the two, they are there as citizens of the full jurisdiction’ (G Mooney, Personal Communication, 29 May 2012). In correspondence to jurors prior to a jury and when briefing jurors, Mooney emphasized ‘you are here as citizens,… of course your family health experiences will be in your head but we don't want to hear about it. You are here representing the community, these are *citizens’* juries’ (G Mooney, Personal Communication, 29 May 2012). In an Australian jury facilitated by Mooney, jurors responded by silencing the personal experience of other jurors as shown in this quote by a juror: ‘You're here as a citizen of [the District], you're not supposed to be talking about your local hospital’ (G Mooney, Personal Communication, 29 May 2012).

Even without explicit direction, jurors themselves often reach a tacit agreement to crowd out personal narratives. One study, which examined the minutiae of the first four UK Citizens’ Councils run by the National Institute for Health and Care Excellence,^6^ identified that jurors ‘use of personal narratives to make points was widespread’.^20^ They observed the potential of personal narratives to ‘bring arguments alive’ 44 by producing ‘a run of similar stories and sometimes an exchange leading to a recognized consensus’.^6^ However, the use of personal narratives in the public deliberation setting was also ‘fraught with difficulty’.^42^ The researchers found that tacit understandings and norms emerged amongst participants about the inappropriateness of sharing personal narratives. They observed a devaluing, ‘censoring and sanctioning of the personal’ which had the potential to undermine deliberation.^6^, ^20^ Participants in their study valued and respected personal stories from expert witnesses but tended to de‐value the personal stories of jurors as being inappropriate in the professional world of a jury setting. Similar findings have been reported from studies of online public deliberation in the United States.^43^, ^44^

Early research on health‐care citizens’ juries in the UK acknowledged a distinction between citizen and consumer perspectives that ‘a person's views may change, depending on whether they are speaking as a service user or a citizen’ and that ‘the aims of consumerism and those of participative democracy may not always be the same’.^21^ One critical finding of an evaluation of early health‐care citizens’ juries in the UK was that ‘the most consistent critics of the citizens’ jury’ were members of local consumer groups, known at the time as Community Health Councils.^21^ A significant concern voiced by those critics was that citizens’ juries may be treated as a public relations exercise, or ‘managed consumerism’, with health authorities influencing the jury's decision by setting the question for the jury and selecting witnesses without (or with token) consumer input.^21^ Others who have written about juries have also made salient points pertaining to the legitimacy of juries as a vehicle for consumer voice, acknowledging the potential for jurors to be confused about their role when sitting on a citizens’ jury.^8^

The potential significance of juror consumer voices on a jury verdict was suggested by a comparative analysis of two juries in the UK, suggesting that personal experience with the health issue in question may have led jurors to a more empathic approach to consumer witnesses, leading to a different line of questioning.^45^ Where jurors had no personal experience with the topic, the lifestyle and testimony of the consumer witness was judged negatively.^45^ Clearly, there is a need for more knowledge about how consumer voices are expressed by jurors and heard by other jurors, what conditions lead to their emergence (or absence) and what contribution those voices have on the deliberative process.

## The artificial distinction between ‘citizen’ and ‘consumer’

The implication of encouraging jurors to participate in juries ‘as citizens, rather than individual users’^8^ is that consumer voices should be silenced. This approach would seem to validate concerns raised by consumer organizations about the citizens’ jury as an effective engagement mechanism. By marginalizing health consumer voices, different experiences and knowledge bases are lost to the jury.^46^ Consumers with diverse experiences may voice different ‘priorities – a distinctive view, another course of action’ that has potential to improve services, reduce system harm and prompt ‘institutional change’ in ways that may not otherwise emerge.^47^

Explicit or implicit expectations that jurors should silence their consumer voices may unfairly marginalize individuals ‘for whom this [exchange of personal stories] is a dominant mode of reasoning and evaluating’.^6^ Indeed, it is argued that public deliberation should be opened up to ‘alternative forms of voice’ and ‘alternative forms of articulating a case’ including ‘story‐telling’.^48^ In contrast, silencing key voices may damage the legitimacy of the deliberative process as a means of engaging the public. As one key thinker in the deliberative democracy field observed:

Legitimate forms of authority and decision making rest on two aspects of deliberative democratic theory: **inclusivity** and the nature of the democratic dialogue. **Inclusivity relates to both presence and voice**: in principle all citizens are entitled to participate in the process of political dialogue and have an equal right to introduce and question claims, to express and challenge needs, values and interests. Voices should not be excluded; parties have an equal right to be heard (our emphasis, Ref. 49).

Lehoux *et al*.^50^ argued that the ‘quest for the ‘ordinary’ citizen is misleading’; rather, the ‘sociological concreteness of citizenship’ should be acknowledged and its implications for deliberation should be understood. Indeed, transparency and explicitness about the views that are brought to the table, and the role of those who bring them, is an important element of procedural justice in health‐care decision making.^51^ Discouragement of jurors’ consumer voices may delimit our understanding of what influenced jurors’ final recommendations *in practice*. Although jurors may be told not to speak of their experiences, personal knowledge may influence their point of view. Unfortunately, the question of whether, how, to what extent, why and with what consequences jurors’ consumer experiences influenced their views is rarely asked.

Clearly ‘the grounds on which citizens are being asked to speak … is fundamental’.^20^ The key principles of effective deliberative public engagement include a requirement that the process involves the right types of people.^52^ But who is the right type and what is their role? Both jurors and witnesses bring the complexity and richness of their lived experience to the jury and are capable of playing ‘a dual role – providing their personal views and representing society’.^41^, ^50^ We need to recognize and understand the mechanisms by which consumer voices and experiences influence the deliberative process, particularly given the rapidly increasing popularity of citizens’ juries.^4^

## Conclusions and future directions

Health services in Australia and elsewhere are currently seeking ways for citizen *and* consumer voices to be heard in the public policymaking process. One method that is gaining prominence is the citizens’ jury. In this paper, we have challenged the assumption that consumer voices are heard in practice.^26^, ^29^, ^53^, ^54^ We have shown multiple gaps in knowledge about the role of consumer voices in citizens’ juries. Research is required to clarify the processes of engaging consumer voices within citizens’ juries including the conditions under which consumer voices can emerge (or not); from whom consumer voices emerge and why; what stories are voiced and when; which voices or stories are silenced and why; and how such voices are heard and with what consequences. The answers to these questions may provide crucial knowledge about the nuances of the deliberative process.

Future empirical research needs to capture ‘the subtleties of deliberation in practice’ 20 to understand the multiple perspectives of multiple jurors across different juries in relation to the phenomenon of consumer voices in such settings. Research of this kind will contribute to an assessment of the usefulness of citizens’ juries as a public engagement mechanism, by illuminating the potential influence of consumer perspectives. It would also reveal insights into the way in which consumer perspectives influence the jury process, as well as whether this influence should or can be avoided. This information will inform the rapidly growing number of stakeholders who are using citizens’ juries to inform their policy decisions.^55^

## Source of funding

Australian Research Council funding LP100200446 and the Australian Centre for Health Services Innovation (PostGraduate Scholarship PHD‐000432).

## Conflicts of interest declaration

The authors are unaware of any potential conflict of interests related to this article.

## Note

1 The term ‘consumer’ refers to lay people who have been, are currently or are likely to be a patient or user of a health service. Use of this term, as opposed to patient or service user, is common in Australia and the United States.^14^ It is acknowledged that the use of the term ‘consumer’ infers a particular position relative to services being provided. However, in our paper, it is the influence of the experience an individual may have had with a health service that is central to our arguments rather than any debate about the use of particular terminology.
